# Opioid-associated iatrogenic withdrawal in critically ill adult patients: a multicenter prospective observational study

**DOI:** 10.1186/s13613-017-0310-5

**Published:** 2017-09-02

**Authors:** Pan Pan Wang, Elaine Huang, Xue Feng, Charles-André Bray, Marc M. Perreault, Philippe Rico, Patrick Bellemare, Paul Murgoi, Céline Gélinas, Annie Lecavalier, Dev Jayaraman, Anne Julie Frenette, David Williamson

**Affiliations:** 1 Pharmacy Department, Lakeshore General Hospital, Montreal, Canada; 2 Pharmacy Department, Hôpital de Verdun, Montreal, Canada; 3 Pharmacy Department, Hôpital du Sacré-Coeur de Montréal, 5400 Gouin West, Montreal, QC H4J 1C5 Canada; 4 Faculté de Pharmacie, Université de Montréal, Montreal, Canada; 5 Pharmacy Department, McGill University Health Center, Montreal, Canada; 6 Critical Care Department, Hôpital du Sacré-Coeur de Montréal, Montreal, Canada; 7 Faculté de Médecine, Université de Montréal, Montreal, Canada; 8 Ingram School of Nursing, McGill University, Montreal, QC Canada; 9 Centre for Nursing Research and Lady Davis Institute, Jewish General Hospital, Montreal, Canada; 10 Department of Adult Critical Care, Jewish General Hospital, McGill University, Montreal, QC Canada; 11 Department of Critical Care, Montreal General Hospital, McGill University Health Center, Montreal, Canada

**Keywords:** Iatrogenic withdrawal syndrome, Opioids, DSM-V, Mechanical ventilation, Critically ill, Intensive care unit, Adult

## Abstract

**Background:**

Opioids and benzodiazepines are frequently used in the intensive care unit (ICU). Regular use and prolonged exposure to opioids in ICU patients followed by abrupt tapering or cessation may lead to iatrogenic withdrawal syndrome (IWS). IWS is well described in pediatrics, but no prospective study has evaluated this syndrome in adult ICU patients. The objective of this study was to determine the incidence of IWS caused by opioids in a critically ill adult population. This multicenter prospective cohort study was conducted at two level-1 trauma ICUs between February 2015 and September 2015 and included 54 critically ill patients. Participants were eligible if they were 18 years and older, mechanically ventilated and had received more than 72 h of regular intermittent or continuous intravenous infusion of opioids. For each enrolled patient and per each opioid weaning episode, presence of IWS was assessed by a qualified ICU physician or senior resident according to the 5th edition of Diagnostic and Statistical Manual of Mental Disorders criteria for opioid withdrawal.

**Results:**

The population consisted mostly of males (74.1%) with a median age of 50 years (25th–75th percentile 38.2–64.5). The median ICU admission APACHE II score was 22 (25th–75th percentile 12.0–28.2). The overall incidence of IWS was 16.7% (95% CI 6–27). The median cumulative opioid dose prior to weaning was higher in patients with IWS (245.7 vs. 169.4 mcg/kg, fentanyl equivalent). Patients with IWS were also exposed to opioids for a longer period of time as compared to patients without IWS (median 151 vs. 125 h). However, these results were not statistically significant.

**Conclusions:**

IWS was occasionally observed in this very specific population of mechanically ventilated, critically ill ICU patients. Further studies are needed to confirm these preliminary results and identify risk factors.

## Background

Opioids and benzodiazepines are frequently used in the intensive care unit (ICU) to treat pain, agitation and facilitate mechanical ventilation [1, 2]. Prolonged stimulation of µ, κ and δ receptors by opioids in the central nervous system and in the peripheral tissues leads to down-regulation of intracellular second-messenger signaling, thus inducing tolerance. If the inhibitory stimulus is abruptly removed, a set of symptoms including central nervous stimulation (e.g., agitation, irritability, tremors, increased wakefulness), sympathetic nervous system hyper-activation (e.g., fever, hypertension, tachycardia, tachypnea, sweating) and gastrointestinal disturbance (e.g., vomiting, nausea, diarrhea) can occur. This phenomenon is known as acute iatrogenic withdrawal syndrome (IWS) [3].

IWS is well described in the pediatric ICU (PICU) population [4–8]. An incidence ranging from 10 to 57% has been reported in children receiving mechanical ventilation and continuous infusion of opioids for more than 24 h [7, 8]. Withdrawal symptoms in the pediatric population, as described by the Finnegan Neonatal Abstinence Scale, include irritability, tremors, clonus, yawning, sneezing, delirium, hypertonicity, seizures and hallucinations [9]. In more severe cases, sympathetic activation may result in tachycardia, hypertension, tachypnea, sweating, fever, as well as gastrointestinal symptoms such as feeding intolerance with vomiting and diarrhea [9, 10]. As a result, IWS can complicate patient recovery [11, 12].

In adult ICU patients, a retrospective study reported an incidence of 32% (9 of 28 patients) for analgesic and sedative medications IWS [13]. In that study, IWS evaluation was based on a modified version of the Himmelsbach scale [13]. To our knowledge, there are no published prospective studies evaluating the incidence and risk factors for opioid-associated IWS in the adult ICU population.

The primary objective of this study was to prospectively evaluate the incidence of IWS in mechanically ventilated adult ICU patients receiving opioids. Possible risk factors for opioid-associated IWS in this patient population were also assessed.

## Methods

### Study design

The study was a prospective observational cohort conducted in two level-1 trauma centers in Montreal, Canada (clinicalTrials.gov. NCT02318290). Enrollment occurred between February 2015 and September 2015. The study was reviewed and approved by the institutional research ethics committees of each participating site.

### Participants

Participants were eligible if they were 18 years and older, mechanically ventilated and had received regular intermittent or continuous intravenous infusion of opioids for more than 72 h. Patients were considered as receiving regular intermittent opioids if more than half of the scheduled “as-needed” doses within the previous 24 h were administered. Initially, participants were included after 96 h of mechanical ventilation and opioids administration. This inclusion criterion was later amended to 72 h due to the limited number of eligible patients. Initial consent was obtained from next of kin, and whenever possible, participation was later confirmed by the patient.

Patients were excluded if they were unable to speak English or French, had physical communication barriers, suffered from severe brain injury defined as Glasgow Coma Scale (GCS) ≤8 or moderate brain injury (GCS 9–12) with elevated intracranial pressure (ICP > 20 mmHg requiring osmotherapy). Other exclusion criteria included imminent and predictable death, active neurological condition such as status epilepticus, encephalopathy, chronic substance abuse (chronic alcohol use defined as ≥2 drinks per day and/or ≥14 drinks per week for men and ≥9 drinks per week for women, regular use of heroin, γ-hydroxybutyric acid, cocaine or amphetamines), chronic use of opioids prior to ICU admission (defined as regular use for a chronic medical reason reported by next of kin or per home medication list), spinal cord injury, and extubation during the first 72 h.

### Procedures and data collection

Patient demographics collected at enrollment included age, gender, past medical history, reason of ICU admission (according to ICD-10 classification) and Acute Physiology and Chronic Health Evaluation II score (APACHE II). Opioids, concomitant sedatives (benzodiazepines, propofol and dexmedetomidine), other co-analgesics, length of ICU stay, and duration of mechanical ventilation were prospectively collected using standardized case report forms. All opioids and benzodiazepines doses were converted into fentanyl and midazolam equivalents, respectively.

There were no standardized opioid weaning protocols at either site. Patient management including all decisions related to analgesia, sedation, weaning and agitation was left to the discretion of the treating team. An opioid weaning episode was defined as a sustained over 4-h ≥10% decrease from the previous stable infusion rate (defined as stable for at least 4 h). Upon weaning, the patient was assessed once daily by an ICU physician to detect the potential development of IWS using the Diagnostic and Statistical Manual 5th edition (DSM-V) criteria for opioid withdrawal [14]. The DSM-V criteria include the presence of either cessation or reduction in opioid use that has been heavy and prolonged (adapted to >72 h in our study) and ≥3 of the following criteria developing within minutes to several days following cessation or reduction: dysphoric mood, nausea or vomiting, muscle aches, lacrimation or rhinorrhea, pupillary dilatation, piloerection, sweating, yawning, fever, insomnia [15]. IWS was diagnosed if ≥3 of the criteria were observed after weaning, and the symptoms could not be explained by another medical condition such as delirium or infection. For each enrolled patient-weaning episode, a second ICU physician or fellow participated in a blinded assessment. A patient was classified as IWS-positive if at least one of the DSM-V evaluations was positive. Patients were followed until death or transfer to another unit. In addition, patients that remained in the ICU were followed for 48 h after the first of the following events: (1) a DSM-V-positive result; (2) an extubation; (3) 14 days after a successful weaning process. Delirium was assessed using daily Confusion Assessment Method for the intensive care unit (CAM-ICU) evaluations.

### Statistical analysis

Descriptive data are expressed as proportions and continuous variables as medians with 25th–75th percentiles. The incidence of IWS is defined as the proportion of patients with a positive IWS diagnosis and is presented with 95% confidence intervals. The Mann–Whitney *U* test was used to compare demographics, cumulative dose of opioids, and duration of exposure to opioids between the IWS-positive and the IWS-negative groups. Chi-square or Fisher’s exact tests, as appropriate, were used to compare exposure to concomitant medications. A two-sided *p* value <0.05 was considered statistically significant. The last observation carried forward (LOCF) was used to analyze missing observations. Listwise deletion was used for missing demographic data. An independent accredited statistician validated the statistical analyses. Data analysis was performed with IBM SPSS Statistics v. 21.0.

## Results

### Patient characteristics

**Fig. 1:**
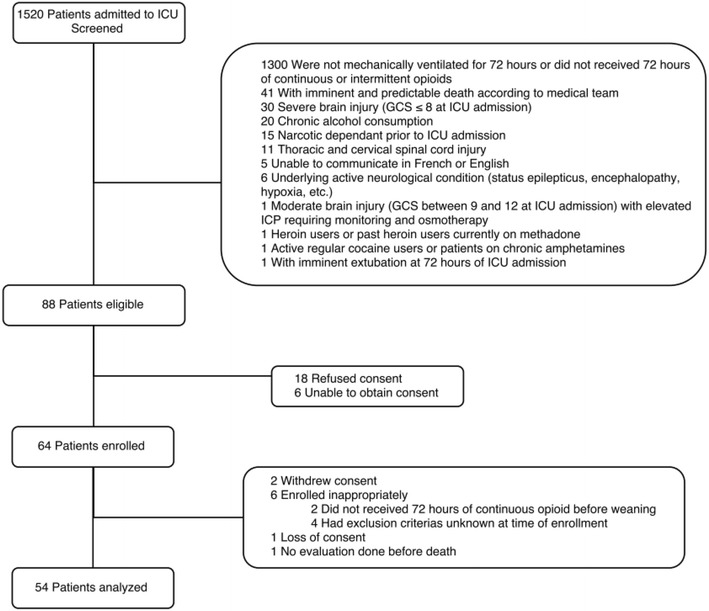
Study flowchart

All patients admitted to ICU within the study period were screened. Of the 1520 patients screened, 54 were included in the study (Fig. 1). Ten and forty-four patients had received opioids for at least 72 and 96 h, respectively. Main reasons for exclusion were short duration of mechanical ventilation, opioid administration less than 72 h (1300 patients) and imminent death (41 patients).

The study population was mainly comprised of men (74.1%) and Caucasians (81.5%) with a median age of 50 years (25th–75th percentile 38.2–64.5) (Table 1). The median APACHE II score was 22.0 (25th–75th percentile 12.0–28.2). The most frequent reasons of admission according to ICD-10 classification were external causes of morbidity (e.g., trauma and injuries) (38.9%) and diseases of the respiratory system (14.8%). Prior to admission, 13 (24.1%) patients reported non-chronic alcohol consumption (<14 drinks per week in men and <9 drinks in women). Thirteen patients (24.1%) were tobacco smokers, and 1 patient (1.9%) was a sporadic recreational amphetamine user. Two patients (3.7%) had received sporadic opioid doses due to acute medical conditions prior to hospital admission.

**Table 1:** Patient demographics

Characteristics	IWS-negative (*n* = 45)	IWS-positive (*n* = 9)	All patients (*n* = 54)
Median age (year) (25th–75th percentile)	53 (40.5–66)	46 (26–59)	50 (38.2–64.5)
Male, no (%)	34 (75.6)	6 (66.3)	40 (74.1)
Median APACHE II (25th–75th percentile)	22 (15.5–28.5)	25 (13–31)	22 (12–28.2)
Median weight (kg) (25th–75th percentile)	85 (79–100)	75 (67.2–95.7)	83.5 (75.8–98.4)
Creatinine ICU adm (μmol/L) (25th–75th percentile)	122 (87–179.5)	106 (72–315)	116 (87–209)
Length of mechanical ventilation (h)	188 (120–358)	286 (197–789.5)	226.5 (124.3–380.3)
Median length of ICU stay, days (IQR)	17 (9.5–22.5)	21 (11–42.5)	17.5 (10–23)
Reason of admission (ICD-10) *n* (%)			
External causes of morbidity (e.g., trauma)	17 (37.8)	4 (44.4)	21 (38.9)
Diseases of the respiratory system	8 (17.8)	0	8 (14.8)
Symptoms and signs not elsewhere classified	4 (8.9)	3 (33.3)	7 (13.0)
Circulatory system	7 (15.6)	0	7 (13.0)
Digestive system	3 (6.7)	0	3 (5.6)
Musculoskeletal/connective tissues	2 (4.4)	1 (11.1)	3 (5.6)
Nervous system	1 (2.2)	1 (11.1)	2 (3.7)
Other categories	3 (6.6)	0 (0)	3 (5.6)

When compared to IWS-negative patients, IWS-positive patients were slightly younger (median age of 46 vs. 53 years; *p* = 0.34), had nonsignificantly higher APACHE II scores (median 25 vs. 22 points; *p* = 0.96), had longer durations of mechanical ventilation (median 286 vs. 188 h; *p* = 0.08) and had longer ICU stays (median 21 vs. 17 days; *p* = 0.21) (Tables 1, 2).

**Table 2:** Opioid exposure and mechanical ventilation

Study group	IWS-negative (*n* = 45) Median (25th–75th percentile) [min, max]	IWS-positive (*n* = 9) Median (25th–75th percentile) [min, max]	*p* value
Cumulative opioid dose prior to weaning (mcg/kg)	169.4 (117,7–234,2) [11.7, 865.6]	245.7 (135,7–437,6) [72.4, 722.4]	*p* = 0.32
Duration of opioid infusion until the first wean (h)^a^	125 (88–243) [57, 564]	151 (81–397) [76, 428]	*p* = 0.47
Duration of mechanical ventilation (h)^b^	188 (122–340) [59, 1201]	286 (207–566) [111, 1245]	*p* = 0.08
Weaning rate (%)	75 (50–100) [12.50, 100]	100 (40–100) [20, 100]	*p* = 0.98

Weaning rate is defined as the difference between the previous stable infusion rate and the new stable infusion rate

^a^The last observation carried forward method was used for 1 patient in each group; opioid begin date = ICU admission date

^b^The last observation carried forward was used for 3 patients in IWS-negative group and 1 in IWS-positive group

### Incidence of IWS

Incidence of IWS was 16.7% (9 out of 54 patients) (95% CI, 6–27%). Onset of IWS ranged from 1 to 11 days following opioid cessation or dose reduction (median = 2 days; 25th–75th percentile 1–4). The agreement between two raters for the 38 evaluations of IWS-based DSM-V criteria was concordant in 90.1% of cases.

### Risk factors for IWS

Although not statistically significant (*p* = 0.32), the cumulative opioid dose (fentanyl equivalent) prior to weaning was greater in IWS-positive (median 245.7 mcg/kg; 25th–75th percentile 135.7–437.6) than in IWS-negative patients (median 169.4 mcg/kg; 25th–75th percentile 117.6–234.2) (Table 2). Likewise, duration of continuous opioid prior to weaning was longer in the IWS-positive group (median 151 h; 25th–75th percentile 81–397) compared to the IWS-negative group (median 125 h; 25th–75th percentile 88–243) (*p* = 0.47). Peak daily opioid dose prior to weaning was also higher in IWS-positive patients (median 4175 mcg; 3130–4997.5 25th–75th percentile) than in IWS-negative patients (3550 mcg; 2737.5–4650 25th–75th percentile). However, this difference was not statistically significant (*p* = 0.24). The percentage in opioid dose reduction at the time of IWS evaluation compared to baseline was similar (*p* = 0.98) between IWS-positive (median 100%; IQR 40–100) and IWS-negative patients (median 75%; IQR 50–100).

Benzodiazepines (100 vs. 71.1%; *p* = 0.254) and clonidine (22.2 vs. 15.6%; *p* = 0.469) were used more frequently in the IWS-positive group than in the IWS-negative group, but it did not reach statistical significance. Antipsychotics (100 vs. 57.8%; *p* = 0.013) were significantly used more frequently in the IWS-positive group than in the IWS-negative group (Table 3). When only considering exposed patients, the benzodiazepine cumulative daily dose was more important in patients diagnosed with IWS (median 12.91 vs. 5.84 mg/kg; *p* = 0.235). In comparison, propofol (97.8 vs. 88.9%; *p* = 0.308) and dexmedetomidine (31.1 vs. 22.2%; *p* = 0.463) were used more frequently in the IWS-negative group.

**Table 3:** Concomitant medications received until end of follow-up

Characteristics	IWS-negative (*n* = 45)	IWS-positive (*n* = 9)
Median cumulative dose of benzodiazepine (mg/kg) (25th–75th percentiles)	5.84 (1.29–13.64) (*n* = 29)	12.91 (3.92–14.10) (*n* = 8)
Propofol	44 (97.8)	8 (88.9)
Dexmedetomidine	14 (31,1)	2 (22.2)
Benzodiazepine	32 (71.1)	9 (100)
Acetaminophen	45 (100)	9 (100)
Pregabalin	5 (11.1)	1 (11.1)
Clonidine	7 (15.6)	2 (22.2)
Antipsychotics	26 (57.8)	9 (100)*

* *p* = <0.05

### Delirium

The overall incidence of delirium during the study was 35.2% (19/54 patients). Delirium was concomitantly identified in 4 of the 9 patients who were identified with IWS (44%).

## Discussion

To our knowledge, this is the first prospective study to evaluate the incidence of IWS in an adult ICU population and to explore its potential risk factors. We reported an IWS incidence of 16.7% (95% CI 6–27%) in our study population. IWS is probably uncommon in the general ICU population. However, it may be more frequent in patients with prolonged mechanical ventilation requiring long-term opioids. Other authors have reported a higher incidence of IWS (32%) [13]. A much shorter duration of mechanical ventilation (12 vs. 39 days in patients with IWS) and a shorter study inclusion opioid exposure (72 vs. 96 h) in our patients could explain this discrepancy [13]. Also, some IWS cases may have been missed because of short-term follow-up. On the other hand, the prospective observational design of this study may have sensitized the physicians to the possible presence of IWS in patients and to its potential prevention. This could potentially have influenced the observed incidence.

In our study, only about 1% of the screened patients were opioid dependent prior to admission. It would be fair to expect opioid withdrawal to be more common in a population with higher rates of opioid use and abuse. Another possible explanation for a lower IWS incidence is our relatively short follow-up for some of the included patients. In the PICU population, withdrawal symptoms have been reported up to 6 days following ≥10% weaning of opioids and/or benzodiazepines [6]. Similarly, in the retrospective study by Cammarano et al., 9 out of 28 patients experienced withdrawal, of which 2 developed IWS in the ICU and 7 on the ward [13]. In our study, the median onset of IWS was 2 days after opioid weaning. However, 25 of the 45 IWS-negative patients (55.6%) were not followed for more than 48 h after extubation as per protocol, and many were rapidly discharged from the ICU to other care units. The later occurrence of IWS in these patients is therefore unknown. As the collaborating physicians were no longer the patients-treating physicians once patients left the ICU, we were unable to prospectively evaluate IWS after ICU discharge.

As previously reported in the PICU population, the most probable risk factors for IWS are the cumulative opioid dose and the duration of continuous exposure to opioids [15]. Although the differences did not reach statistical significance, the median cumulative opioid dose adjusted for weight, the median daily peak dose of opioid and the median duration of opioid exposure were higher in the IWS-positive group than in the IWS-negative group. A pediatric study also identified a rapid opioid dose decrease as a risk factor for IWS [7]. This observation was not confirmed by our study. It was also impossible to distinguish the possible association of IWS with specific opioid agents, since all but 3 patients (6% receiving morphine) were on fentanyl infusions.

In PICU studies, IWS has been associated with increased morbidity, hospital costs and psychological distress [7]. Our data suggest similar associations as patients in the IWS-positive group were more heavily sedated (cumulative benzodiazepine dose 12.91 vs. 5.84 mg/kg), were mechanically ventilated for a longer period and had longer ICU stays.

This study has several strengths including its prospective design. As there is currently no validated tool to identify opioid withdrawal syndrome in the adult ICU population, the diagnosis was performed using the DSM-V criteria for opioid withdrawal. IWS diagnosis was also corroborated by clinical judgment, taking into account other differential diagnosis for the featured symptoms. Systematically using the CAM-ICU, delirium was concomitantly diagnosed in 44% of our patients presenting IWS. However, the overlapping of delirium with IWS remains unstudied.

This study also has several limitations including its small sample size. The agreement between raters analysis for the diagnosis of IWS using the DSM-V was also limited to concordance because of the low prevalence of IWS. The reliability and accuracy of the DSM-V for the diagnosis of IWS in an adult ICU population remain to be studied. While others have studied both opioid- and benzodiazepine-related IWS, we focused on opioid-associated IWS [13, 15]. The administration of benzodiazepines was treated as a potential confounding factor. The specific contribution of benzodiazepine exposure to IWS could not be isolated, as benzodiazepines and opioids are often co-administered. Frequency of benzodiazepine use was not statistically different in patients with and without IWS. However, we cannot exclude the contribution of benzodiazepine to IWS in the exposed patients.

The results of this study can only be extrapolated to a subset of patients admitted to the ICU. Of note, 86% of the initially screened patients were excluded due to the absence of mechanical ventilation or because of insufficient opioid exposure. Finally, 38% of the eligible patients refused or withdrew consent to participate, which also weakens the external validity of the study. In summary, more studies are needed for greater recognition of the syndrome and appropriate prevention of IWS in the adult population.

## Conclusion

IWS is occasionally observed in critically ill adult patients mechanically ventilated and receiving opioids for more than 72 h. Higher cumulative dose and longer exposure of opioid may have contributed to the increased risk of IWS. Future studies with a larger sample size and a longer follow-up period are needed to confirm these preliminary results.

## Abbreviations

APACHE II: Acute Physiology and Chronic Health Evaluation II score
CAM-ICU: Confusion Assessment Method for the intensive care unit
DSM-V: 5th edition of Diagnostic and Statistical Manual of Mental Disorders
ICU: intensive care unit
IWS: iatrogenic withdrawal syndrome
LOCF: last observation carried forward
PICU: pediatric intensive care unit
WAAICUP-1: Withdrawal Assessment in Adult ICU Population-1
